# Refractory cardiac arrest caused by type I Kounis syndrome treated with adrenaline and nicorandil: A case report

**DOI:** 10.1097/MD.0000000000034535

**Published:** 2023-08-11

**Authors:** Taiga Ichinomiya, Motohiro Sekino, Megumi Toba, Akihiro Yokoyama, Naoya Iwasaki, Yusuke Kasai, Hiroshi Araki, Rintaro Yano, Sojiro Matsumoto, Masaya Kurobe, Ryu Sasaki, Tetsuya Hara

**Affiliations:** a Department of Anesthesiology and Intensive Care Medicine, Nagasaki University Graduate School of Biomedical Sciences, Nagasaki, Japan; b Department of Anesthesia, Sasebo City General Hospital, Nagasaki, Japan; c Department of Cardiovascular Medicine, Nagasaki University Graduate School of Biomedical Sciences, Nagasaki, Japan; d Department of Gastroenterology and Hepatology, Nagasaki University Graduate School of Biomedical Sciences, Nagasaki, Japan.

**Keywords:** acute coronary syndrome, anaphylactic shock, anaphylaxis, cardiopulmonary arrest, coronary vasodilator, coronary vasospasm, epinephrine

## Abstract

Kounis syndrome is a rare but life-threatening anaphylactic reaction that can lead to acute coronary syndrome and cardiac arrest, and requires prompt diagnosis. Adrenaline, which is used to treat anaphylaxis, may cause coronary vasoconstriction and worsen ischemia, whereas coronary vasodilators may dilate systemic vessels and exacerbate hypotension. Delayed diagnosis of Kounis syndrome and inadequate therapeutic intervention may thus lead to a poor outcome.

**Patient concerns:**

A 59-year-old man was treated for sepsis due to a liver abscess. Following administration of daptomycin, the patient developed severe anaphylactic shock leading to refractory cardiac arrest. Because conventional cardiopulmonary resuscitation was ineffective, extracorporeal cardiopulmonary resuscitation was considered as an alternative approach.

**Diagnoses:**

On bedside monitoring during cardiopulmonary resuscitation, unexpected ST-segment elevation was found on lead II electrocardiogram. Accordingly, the patient was clinically diagnosed with Kounis syndrome.

**Interventions:**

Nicorandil (6 mg/h), a coronary vasodilator with minimal blood pressure effects, was administered along with high doses of vasopressors, including adrenaline 0.2 µg/kg/min.

**Outcomes:**

After the initiation of nicorandil administration, the patient achieved return of spontaneous circulation and did not require extracorporeal cardiopulmonary resuscitation. Based on the elevated serum tryptase level, normal creatine kinase-MB range, and lack of stenosis on coronary angiography, the patient was definitively diagnosed with type I (coronary vasospasm) Kounis syndrome. He was subsequently transferred to the referring hospital without neurological sequelae.

**Lessons:**

If anaphylaxis leads to refractory shock and cardiac arrest, ischemic changes on the electrocardiogram should be investigated to identify underlying Kounis syndrome. In addition to adrenaline, coronary dilators are the definitive treatment. Nicorandil may be a useful treatment option because of its minimal effect on blood pressure.

## 1. Introduction

Kounis syndrome is an acute coronary syndrome (ACS) caused by an increase in several inflammatory mediators, such as biogenic amines, including histamine; neutral proteases, including tryptase; and arachidonic acid products, upon an anaphylactic response.^[1]^ It is classified into 3 types: coronary vasospasm (type I), acute myocardial infarction (type II), and stent thrombosis (type III), accounting for 73%, 22%, and 5% of case reports, respectively.^[1,2]^

The incidence of Kounis syndrome in the emergency department has been reported to be 0.02% of all admissions and 3.4% of all allergy patients,^[1]^ suggesting that it is a rare disease. However, Kounis syndrome has also been described as an underdiagnosed disease due to its broad spectrum of clinical symptoms, signs, and findings caused by anaphylaxis and myocardial ischemia.^[1,2]^ Thus, the actual incidence may be much higher.

In contrast, the mortality rate associated with Kounis syndrome is as high as 7.0%,^[3]^ and prompt diagnosis and appropriate treatment are necessary to improve prognosis. However, the recent clinical practice guidelines for anaphylaxis do not mention Kounis syndrome or its diagnosis,^[4–7]^ particularly the importance of ischemic changes on electrocardiography (ECG). On the other hand, with regard to the treatment, the use of adrenaline to treat anaphylaxis may cause coronary vasoconstriction, worsening ischemia.^[1,8]^ Conversely, coronary vasodilators, such as nitroglycerin and calcium channel blockers, may dilate systemic vessels and worsen hemodynamic instability.^[1]^ Therefor, there are still concerns about the diagnosis and treatment of Kounis syndrome in clinical practice.

Here, we report a case of refractory cardiac arrest diagnosed as Kounis syndrome based on ST-segment elevation on lead II of the bedside ECG, which improved markedly after the administration of nicorandil, a coronary vasodilator, in addition to adrenaline.

## 2. Case presentation

A 59-year-old man (height, 165 cm; weight, 83 kg) was treated for a liver abscess at another hospital but was transferred to our hospital because of sepsis. His medical history included diabetes mellitus and hyperlipidemia, and he was not taking any β-blockers. The patient exhibited mild disturbance of consciousness, deteriorated oxygenation, acute kidney injury, and disseminated intravascular coagulation due to sepsis.

After percutaneous liver abscess drainage, he was admitted to the intensive care unit (ICU), where antibiotics (daptomycin, meropenem, and metronidazole) were administered. However, after daptomycin administration, the patient experienced severe agitation and his respiratory condition suddenly deteriorated, and mechanical ventilation was initiated. There were no signs of bronchospasm or abnormal skin findings, but fluid resuscitation and noradrenaline (maximum 0.3 µg/kg/min) were required. We diagnosed the patient with septic shock and started continuous renal replacement therapy for acute kidney injury as an adjunctive therapy. Hemodynamics improved over time and the dose of noradrenaline was reduced to 0.05 µg/kg/min by the next day.

**Figure 1.**
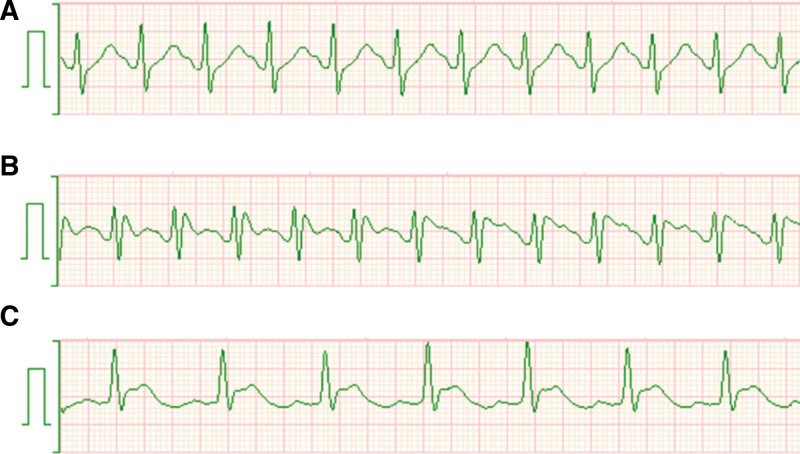
Lead II of the electrocardiogram on the bedside monitor. There was no abnormality in the ST-segment of the lead II immediately after resuscitation from pulseless electrical activity (A). However, when the blood pressure began to decrease again, ST-segment elevation was observed (B). Shortly thereafter, the patient went into ventricular fibrillation. Immediately after return of spontaneous circulation, improvement of ST-segment elevation was observed (C).

About 24 hours after ICU admission (20 minutes after the second daptomycin administration), the patient suddenly went into shock again. Fluid resuscitation and noradrenaline administration (0.5 µg/kg/min) were ineffective, and the patient finally developed pulseless electrical activity. Chest compressions were initiated, and 1 mg of adrenaline was injected intravenously. Subsequently, systolic blood pressure (BP) temporarily increased to >100 mm Hg, at which time he experienced generalized erythema, leading to suspicion of anaphylactic shock. At that time, no abnormalities in the ST-segment of lead II ECG on bedside monitoring were noted (Fig. 1A). Systolic BP again decreased to <80 mm Hg, resulting in ventricular fibrillation. Four additional 1-mg doses of adrenaline and a 300-mg dose of amiodarone were administered intravenously, after which administration of 0.2 µg/kg/min of adrenaline was initiated. Electrical defibrillation was performed twice but was ineffective. In addition, ST-segment elevation in lead II of the ECG was observed immediately before the start of ventricular fibrillation (Fig. 1B). The patient was clinically diagnosed with Kounis syndrome, and nicorandil (Sawai Pharmaceutical Co., Ltd., Osaka, Japan) was started at 6 mg/h (the maximum dose for unstable angina stated in the package insert). Extracorporeal cardiopulmonary resuscitation was considered. However, after the administration of nicorandil, return of spontaneous circulation (ROSC) was achieved by electrical defibrillation. The duration of cardiac arrest was 18 minutes. Immediately after ROSC, an improvement in the ST-segment elevation on lead II ECG was observed (Fig. 1C). Although high doses of vasopressors (adrenaline 0.2 µg/kg/min, noradrenaline 0.7 µg/kg/min, and vasopressin 1.8 U/h) were temporarily required, hemodynamics improved over time. A 100-mg hydrocortisone bolus and continuous hydrocortisone infusion 200 mg/day were started to improve hemodynamics and prevent a delayed anaphylactic reaction. Four hours after resuscitation, BP could be maintained with 0.05 µg/kg/min of noradrenaline and 0.05 µg/kg/min of adrenaline.

**Figure 2.**
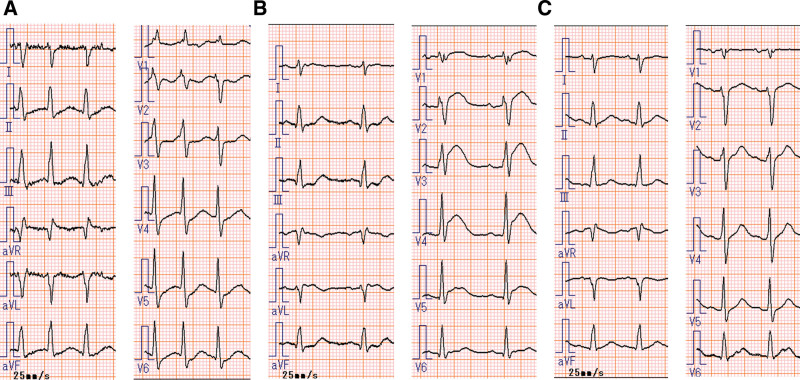
Compared to the electrocardiogram upon intensive care unit admission (A), 12-lead electrocardiogram 10 minutes after return of spontaneous circulation (B) revealed V2–5 ST-segment elevation. However, these changes returned to normal 30 minutes after return of spontaneous circulation (C).

Twelve-lead ECG at 10 minutes after ROSC (Fig. 2B) revealed V2–5 ST-segment elevation as compared to that at ICU admission (Fig. 2A), but these changes returned to their normal levels 30 minutes after ROSC (Fig. 2C). Transthoracic echocardiography revealed no abnormalities in regional wall motion. The creatine kinase-MB level measured 12 hours after the event was 3 U/L (within the normal range). Furthermore, anaphylaxis, ECG changes, and cardiovascular events did not recur. Adrenaline was discontinued 60 hours after the anaphylactic event.

**Figure 3.**
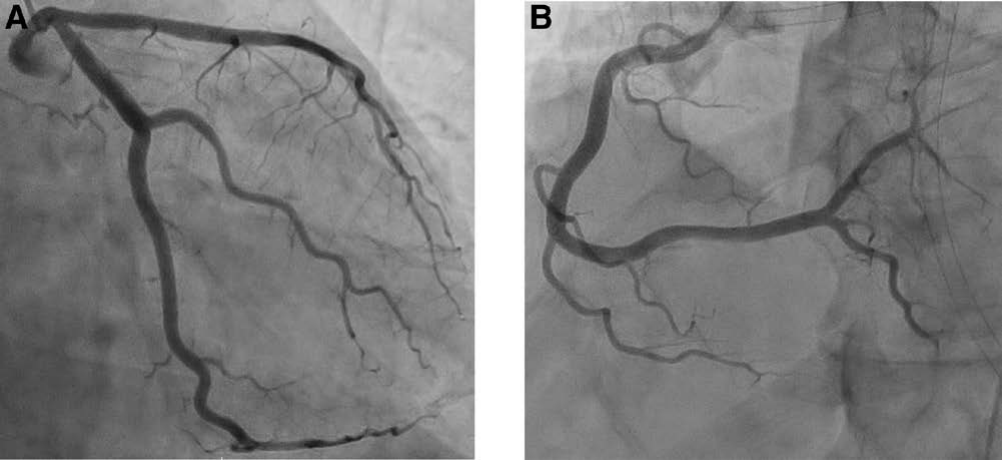
Coronary angiography revealed no abnormal findings in the left (A) or right (B) coronary arteries.

On day 8 after admission, the tracheal tube was removed, and coronary angiography showed no coronary obstruction (Fig. 3A and B). Furthermore, the serum tryptase level at the time of the anaphylactic event, which was later determined, was significantly elevated to 128 mg/mL (normal range: 2.1–9.0 mg/mL). Based on these findings, we concluded that the patient’s cardiac arrest was due to type I Kounis syndrome. The fact that the second dose of daptomycin was administered alone 20 minutes before the anaphylactic shock occurred, as well as that the sudden deterioration occurred after the first dose of daptomycin 24 hours earlier, suggested that daptomycin was the causative agent.

The patient was discharged from the ICU on day 12 and was transferred to the referring hospital on day 39 for continued treatment of the liver abscess, after which he was discharged without neurological sequelae.

## 3. Discussion

The mortality rate of Kounis syndrome is as high as 7.0%,^[3]^ which is significantly higher than the 0.3% to 0.4% mortality rate for anaphylaxis patients requiring hospitalization.^[3,9]^ According to a previous report, about half of the Kounis syndrome patients who experience cardiac arrest, die.^[2]^ Medical drugs such as antibiotics are the most common triggers for “Kounis syndrome”^[1,2,10]^ and “refractory anaphylactic shock.”^[11–14]^ These are iatrogenic complications requiring prompt diagnosis and appropriate treatment by healthcare providers involved in the administration of the causative agents.

Kounis syndrome is comprehensively diagnosed based on clinical symptoms and findings from ECG, echocardiography, angiography, and laboratory tests.^[1]^ In addition to anaphylactic symptoms, chest pain (87%) and ST-segment abnormalities (93%) on ECG are common clinical presentations.^[2]^ However, when the patient is in a fatal condition or under sedation, diagnosis must be made clinically and quickly, based on limited information. Taken together, anaphylaxis symptoms, including hemodynamic instability after exposure to an allergen, and ST-segment abnormalities on ECG play important roles in rapid diagnosis.^[10]^ In the present case, the diagnosis was made based on ST-segment elevation in lead II on the bedside ECG monitor, but lead II can only detect myocardial ischemia in a limited region. Three-channel ECGs on bedside monitors can evaluate leads I, II, III, aVR, aVL, and aVF by switching leads. However, the detection rate of myocardial ischemia is 20% inferior to that of 12-lead ECGs.^[15]^ Depending on the situation, it is important to aggressively evaluate myocardial ischemia using a bedside monitor ECG or 12-lead ECG, if available. However, recent anaphylaxis guidelines do not mention Kounis syndrome or the importance of ECG in its diagnosis.^[4–7]^ To date, only Japanese perioperative anaphylaxis guidelines have mentioned it.^[16]^ It is desirable to include this information more broadly in guidelines to facilitate early diagnosis and treatment.

With regard to treatment, adrenaline is the first-line therapy for anaphylaxis and anaphylactic shock. This is because it inhibits the release of histamine and other anaphylactic mediators, and it has strong stimulating effects on α- and β-receptors.^[17]^ However, adrenaline may also exacerbates myocardial ischemia by further reducing coronary blood flow that is already impaired in Kounis syndrome due to factors such as coronary vasospasm, thrombus formation, and tachycardia.^[1,8,18,19]^ Noradrenaline, vasopressin, and glucagon can be used in addition to adrenaline for treatment-resistant anaphylactic shock,^[4,7,16]^ but there is no evidence to support their use in the absence of adrenaline. Therefore, we believe that, in cases of fatal anaphylaxis and anaphylactic shock, including Kounis syndrome, the use of adrenaline should not be avoided.^[8,20]^ However, hemodynamics should be carefully monitored (particularly by using ECG) to prevent myocardial ischemia. In addition, attention should be given to the dosage and method of administration to minimize the adverse effects of adrenaline.^[19,20]^ Finally, if adrenaline and other vasopressors prove to be ineffective, extracorporeal cardiopulmonary resuscitation should be considered; however, this is an invasive technique and should be avoided if possible.

Coronary vasodilators, such as nitroglycerin and calcium channel blockers are effective in treating patients with ACS, particularly in those with coronary vasospasm. However, their effect on systemic vasodilation may promote hypotension (nitroglycerin and calcium channel blockers) and tachycardia (nitroglycerin); these effects can potentially worsen myocardial ischemia in patients with Kounis syndrome.^[1]^ Thus, the use of these vasodilators should be considered when BP is maintained,^[1]^ but is not recommended in life-threatening situations, such as the present case. Nicorandil, on the other hand, is a coronary vasodilator used in ACS and heart failure cases. It dilates large and small coronary arteries through nitrate-like and potassium channel-opening effects, but is less likely to lower BP in patients with low to normal BP.^[21–24]^ Nicorandil can be administered as a bolus or loading dose, as well as by continuous infusion, although there is only 1 successful case report of its use as a bolus in Kounis syndrome, in a patient with coronary vasospasm and hypotension.^[25]^ Several other case reports have described using nicorandil in Kounis syndrome. In all of these cases, continuous infusion was employed, and the patients had relatively stable hemodynamic status, or received the drug prophylactically;^[20,26–28]^ None of these cases involved administering nicorandil in a patient with cardiac arrest status, as in the present case. The efficacy of nicorandil in fatal conditions, such as refractory shock and cardiac arrest, as well as the method of administration and dosage, require further investigation. However, it has favorable pharmacological features and is expected to be a useful drug.^[10,29]^

Fortunately, in this case, the patient was diagnosed with Kounis syndrome based on ST-segment elevation in lead II on the bedside ECG monitor. The ST-segment elevation improved markedly upon administration of nicorandil and adrenaline. If the abnormal ECG had not been noticed and nicorandil had not been administered, ROSC would not have been achieved. Therefore, it is important to emphasize the need to evaluate myocardial ischemia in patients with suspected anaphylaxis as a standard practice in clinical settings. Furthermore, the efficacy of nicorandil, particularly in cases of severe hypotension and cardiac arrest, as well as the appropriate dosage and method of administration, need to be further evaluated through case accumulation.

In conclusion, we have described a case of refractory cardiac arrest that was diagnosed as Kounis syndrome based on ST-segment elevation in lead II on bedside ECG. ST-segment elevation improved markedly after administration of nicorandil. This case highlights the importance of conducting an aggressive evaluation of myocardial ischemia by ECG in patients with anaphylaxis. In addition to using adrenaline as a first-line treatment, nicorandil may improve the prognosis of patients with Kounis syndrome.

## Acknowledgments

We would like to thank Editage (www.editage.jp) for English language editing.

## Author contributions

**Conceptualization:** Taiga Ichinomiya, Motohiro Sekino, Megumi Toba, Akihiro Yokoyama, Naoya Iwasaki, Yusuke Kasai, Hiroshi Araki, Sojiro Matsumoto, Masaya Kurobe, Ryu Sasaki, Tetsuya Hara.

**Investigation:** Taiga Ichinomiya, Motohiro Sekino, Megumi Toba, Akihiro Yokoyama, Naoya Iwasaki, Yusuke Kasai, Hiroshi Araki, Rintaro Yano, Sojiro Matsumoto, Masaya Kurobe, Ryu Sasaki, Tetsuya Hara.

**Supervision:** Motohiro Sekino, Tetsuya Hara.

**Visualization:** Taiga Ichinomiya.

**Writing – original draft:** Taiga Ichinomiya.

**Writing – review & editing:** Motohiro Sekino, Megumi Toba, Akihiro Yokoyama, Naoya Iwasaki, Yusuke Kasai, Hiroshi Araki, Rintaro Yano, Sojiro Matsumoto, Masaya Kurobe, Ryu Sasaki, Tetsuya Hara.

## References

[1] Kounis NG. Kounis syndrome: an update on epidemiology, pathogenesis, diagnosis and therapeutic management. Clin Chem Lab Med. 2016;54:1545–59. 26966931 10.1515/cclm-2016-0010

[2] Abdelghany M Subedi R Shah S. Kounis syndrome: a review article on epidemiology, diagnostic findings, management and complications of allergic acute coronary syndrome. Int J Cardiol. 2017;232:1–4. 28153536 10.1016/j.ijcard.2017.01.124

[3] Desai R Parekh T Patel U. Epidemiology of acute coronary syndrome co-existent with allergic/hypersensitivity/anaphylactic reactions (Kounis syndrome) in the United States: a nationwide inpatient analysis. Int J Cardiol. 2019;292:35–8. 31204069 10.1016/j.ijcard.2019.06.002

[4] Li X Ma Q Yin J. A clinical practice guideline for the emergency management of anaphylaxis (2020). Front Pharmacol. 2022;13:845689. 35418863 10.3389/fphar.2022.845689 PMC8996305

[5] Cardona V Ansotegui IJ Ebisawa M. World allergy organization anaphylaxis guidance 2020. World Allergy Organ J. 2020;13:100472. 33204386 10.1016/j.waojou.2020.100472 PMC7607509

[6] Muraro A Worm M Alviani C. EAACI guidelines: anaphylaxis (2021 update). Allergy. 2022;77:357–77. 34343358 10.1111/all.15032

[7] Garvey LH Dewachter P Hepner DL. Management of suspected immediate perioperative allergic reactions: an international overview and consensus recommendations. Br J Anaesth. 2019;123:e50–64. 31130272 10.1016/j.bja.2019.04.044

[8] Urushidani S Kuriyama A. A potential association between myocardial ischemia and epinephrine for anaphylaxis. Am J Emerg Med. 2020;38:1297.e1–3. 10.1016/j.ajem.2020.01.033 31983596

[9] Ma L Danoff TM Borish L. Case fatality and population mortality associated with anaphylaxis in the United States. J Allergy Clin Immunol. 2014;133:1075–83. 24332862 10.1016/j.jaci.2013.10.029 PMC3972293

[10] Dai B Cavaye J Judd M. Perioperative presentations of Kounis syndrome: a systematic literature review. J Cardiothorac Vasc Anesth. 2022;36:2070–6. 35260322 10.1053/j.jvca.2022.01.042

[11] Clark S Wei W Rudders SA. Risk factors for severe anaphylaxis in patients receiving anaphylaxis treatment in US emergency departments and hospitals. J Allergy Clin Immunol. 2014;134:1125–30. 24985399 10.1016/j.jaci.2014.05.018

[12] Bilò MB Corsi A Martini M. Fatal anaphylaxis in Italy: analysis of cause-of-death national data, 2004-2016. Allergy. 2020;75:2644–52. 32364284 10.1111/all.14352

[13] Turner PJ Jerschow E Umasunthar T. Fatal anaphylaxis: mortality rate and risk factors. J Allergy Clin Immunol Pract. 2017;5:1169–78. 28888247 10.1016/j.jaip.2017.06.031 PMC5589409

[14] Park H Kim SM Kim WY. Cardiac arrest caused by anaphylaxis refractory to prompt management. Am J Emerg Med. 2022;61:74–80. 36057212 10.1016/j.ajem.2022.08.035

[15] Loewe A Schulze WH Jiang Y. ECG-based detection of early myocardial ischemia in a computational model: impact of additional electrodes, optimal placement, and a new feature for ST deviation. Biomed Res Int. 2015;2015:530352. 26587538 10.1155/2015/530352 PMC4637443

[16] Takazawa T Yamaura K Hara T. Practical guidelines for the response to perioperative anaphylaxis. J Anesth. 2021;35:778–93. 34651257 10.1007/s00540-021-03005-8

[17] Kemp SF Lockey RF Simons FE. Epinephrine: the drug of choice for anaphylaxis. A statement of the world allergy organization. Allergy. 2008;63:1061–70. 18691308 10.1111/j.1398-9995.2008.01733.x

[18] Yasue H Touyama M Kato H. Prinzmetal’s variant form of angina as a manifestation of alpha-adrenergic receptor-mediated coronary artery spasm: documentation by coronary arteriography. Am Heart J. 1976;91:148–55. 813507 10.1016/s0002-8703(76)80568-6

[19] Yokoyama A Sekino M Ichinomiya T. Anaphylactic shock in a patient with severe aortic stenosis treated with adrenaline and landiolol for circulatory management: a case report. Med (Baltim). 2021;100:e27135. 10.1097/MD.0000000000027135 PMC8416007 34477163

[20] Shintani R Sekino M Egashira T. Allergen-related coronary vasospasm “Kounis syndrome” requiring administration of epinephrine and a coronary vasodilator. J Cardiothorac Vasc Anesth. 2021;35:2768–71. 32888803 10.1053/j.jvca.2020.08.009

[21] Nakae I Matsumoto T Horie H. Effects of intravenous nicorandil on coronary circulation in humans: plasma concentration and action mechanism. J Cardiovasc Pharmacol. 2000;35:919–25. 10836727 10.1097/00005344-200006000-00014

[22] Minami Y Nagashima M Kajimoto K. Acute efficacy and safety of intravenous administration of nicorandil in patients with acute heart failure syndromes: usefulness of noninvasive echocardiographic hemodynamic evaluation. J Cardiovasc Pharmacol. 2009;54:335–40. 19687747 10.1097/FJC.0b013e3181b76730

[23] Tanaka K Kato K Takano T. Acute effects of intravenous nicorandil on hemodynamics in patients hospitalized with acute decompensated heart failure. J Cardiol. 2010;56:291–9. 20709498 10.1016/j.jjcc.2010.06.009

[24] Qian G Zhang Y Dong W. Effects of nicorandil administration on infarct size in patients with ST-segment-elevation myocardial infarction undergoing primary percutaneous coronary intervention: the CHANGE trial. J Am Heart Assoc. 2022;11:e026232. 36073634 10.1161/JAHA.122.026232 PMC9683654

[25] Adachi H Ihara M Nojima Y. Kounis syndrome caused by anaphylaxis without skin manifestations after cefazolin administration. J Allergy Clin Immunol Pract. 2019;7:317–9. 29902529 10.1016/j.jaip.2018.05.030

[26] Yanai M Ariyoshi K. Two cardiac arrests that occurred after the administration of sugammadex: a case of Kounis syndrome. Case Rep Emerg Med. 2020;2020:6590101. 10.1155/2020/6590101 PMC7048914 32128264

[27] Okuno A Matsuki Y Tabata M. A suspected case of coronary vasospasm induced by anaphylactic shock caused by rocuronium-sugammadex complex. J Clin Anesth. 2018;48:7. 29660702 10.1016/j.jclinane.2018.03.017

[28] Sato M Arai T. A case of Kounis syndrome presenting as coronary artery spasm associated with cefazolin-induced anaphylaxis during general anesthesia. JA Clin Rep. 2019;5:49. 32026020 10.1186/s40981-019-0269-3 PMC6966758

[29] Akita T Kawata M Sakaguchi A. Successful treatment of prolonged cardiopulmonary arrest of Kounis syndrome during coronary angioplasty. J Cardiol Cases. 2016;13:47–51. 30524554 10.1016/j.jccase.2015.10.001 PMC6262128

